# SNPs Associated with Cerebrospinal Fluid Phospho-Tau Levels Influence Rate of Decline in Alzheimer's Disease

**DOI:** 10.1371/journal.pgen.1001101

**Published:** 2010-09-16

**Authors:** Carlos Cruchaga, John S. K. Kauwe, Kevin Mayo, Noah Spiegel, Sarah Bertelsen, Petra Nowotny, Aarti R. Shah, Richard Abraham, Paul Hollingworth, Denise Harold, Michael M. Owen, Julie Williams, Simon Lovestone, Elaine R. Peskind, Ge Li, James B. Leverenz, Douglas Galasko, The Alzheimer's Disease Neuroimaging Initiative, John C. Morris, Anne M. Fagan, David M. Holtzman, Alison M. Goate

**Affiliations:** 1 Department of Psychiatry, Washington University School of Medicine, St. Louis, Missouri, United States of America; 2 The Hope Center Program on Protein Aggregation and Neurodegeneration (HPAN), Washington University School of Medicine, St. Louis, Missouri, United States of America; 3 Department of Biology, Brigham Young University, Provo, Utah, United States of America; 4 Department of Neurology, Washington University School of Medicine, St. Louis, Missouri, United States of America; 5 Department of Psychological Medicine, Medical Research Council (MRC) Centre for Neuropsychiatric Genetics and Genomics, School of Medicine, Cardiff University, Cardiff, United Kingdom; 6 Kings College, London, United Kingdom; 7 Departments of Psychiatry and Behavioral Sciences, University of Washington School of Medicine, Seattle, Washington, United States of America; 8 Veterans Affairs Northwest Network Mental Illness Research, Education, and Clinical Center, Seattle, Washington, United States of America; 9 Department of Neurology, University of Washington School of Medicine, Seattle, Washington, United States of America; 10 Department of Neurosciences, University of California San Diego, La Jolla, California, United States of America; 11 Department of Pathology and Immunology, Washington University School of Medicine, St. Louis, Missouri, United States of America; 12 Department of Developmental Biology, Washington University School of Medicine, St. Louis, Missouri, United States of America; 13 Alzheimer's Disease Research Center, Washington University School of Medicine, St. Louis, Missouri, United States of America; University of Miami, Miller School of Medicine, United States of America

## Abstract

Alzheimer's Disease (AD) is a complex and multifactorial disease. While large genome-wide association studies have had some success in identifying novel genetic risk factors for AD, case-control studies are less likely to uncover genetic factors that influence progression of disease. An alternative approach to identifying genetic risk for AD is the use of quantitative traits or endophenotypes. The use of endophenotypes has proven to be an effective strategy, implicating genetic risk factors in several diseases, including anemia, osteoporosis and heart disease. In this study we identify a genetic factor associated with the rate of decline in AD patients and present a methodology for identification of other such factors. We have used an established biomarker for AD, cerebrospinal fluid (CSF) tau phosphorylated at threonine 181 (ptau_181_) levels as an endophenotype for AD, identifying a SNP, rs1868402, in the gene encoding the regulatory sub-unit of protein phosphatase B, associated with CSF ptau_181_ levels in two independent CSF series 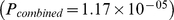. We show no association of rs1868402 with risk for AD or age at onset, but detected a very significant association with rate of progression of disease that is consistent in two independent series 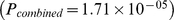. Our analyses suggest that genetic variants associated with CSF ptau_181_ levels may have a greater impact on rate of progression, while genetic variants such as *APOE4*, that are associated with CSF Aβ_42_ levels influence risk and onset but not the rate of progression. Our results also suggest that drugs that inhibit or decrease tau phosphorylation may slow cognitive decline in individuals with very mild dementia or delay the appearance of memory problems in elderly individuals with low CSF Aβ_42_ levels. Finally, we believe genome-wide association studies of CSF tau/ptau_181_ levels should identify novel genetic variants which will likely influence rate of progression of AD.

## Author Summary

Alzheimer's disease (AD) is the most common neurodegenerative disease affecting more than 4.5 million people in the US. Genetic studies of AD have previously identified pathogenic mutations in three genes (*APP*, *PSEN1* and *PSEN2*) and polymorphisms in *APOE* as risk factors. These findings have led to a better understanding of the underlying disease mechanisms. However, half of all AD cases have no known genetic risk factors for disease. Most studies are designed to identify variants associated with risk or age at onset, but rarely cover other important facets of AD, such as disease progression or duration. In this study we have used an established AD biomarker (cerebrospinal fluid tau phosphorylated at threonine 181, ptau_181_) to find genetic variants that influence levels of ptau_181_ in the cerebrospinal fluid. This novel and powerful approach has allowed us to identify a genetic factor located in the regulatory subunit of the calcineurin that is also strongly associated with rate of progression of AD. This study is important because it defines a strategy to find novel genetic factors influencing different facets of AD pathobiology including risk, onset and progression.

## Introduction

Genetic studies have helped to further our understanding of the pathogenic mechanism of several diseases, including AD. To date only the ε4 allele of apolipoprotein E (*APOE4*), present in 50% of late onset AD (LOAD) cases, has been convincingly demonstrated to influence risk for LOAD. The traditional method for searching for genetic risk factors involves the comparison of genes in AD cases and non-demented elderly controls. AD is a complex and multifactorial disease, and as a result very large datasets have been necessary to identify these genetic risk factors [1]–[2]. An alternative to the standard case-control study design is to use quantitative traits or endophenotypes. Quantitative traits have been used to successfully identify new genetic factors implicated in anemia [3]–[6], osteoporosis [7] and heart disease [8]–[11]. The advantages of quantitative traits are that they provide higher power than regular case-control analyses, a biological model of disease and the possible effects of the associated genetic variation and may decrease the clinical heterogeneity of the samples. This is likely to be true for Alzheimer's Disease (AD) because up to 30% of individuals in screened elderly non-demented control samples show evidence of AD pathology at autopsy [12], and a similar number have biomarker profiles consistent with preclinical AD [13]–[15], thus reducing the power of a case-control design.

Both Aβ and tau protein play an important role in AD, are detectable in cerebrospinal fluid (CSF) in all individuals, and have been used as biomarkers for diagnosis [16]–[18]. Patients with AD show lower CSF Aβ_42_ levels [19] that inversely correlate with the presence of fibrillar Aβ in the brain (as measured by Pittsburgh Compound B (PET-PIB) retention) in demented individuals [14] and plaque counts in brain samples [20]. Several studies suggest that PET-PIB retention and CSF Aβ_42_ levels could help to identify individuals with AD pathology before the onset of clinically detectable disease (preclinical AD) [14], [21]. The CSF levels of total tau and tau phosphorylated at threonine 181 (ptau_181_) are increased in AD [12], [14]. Elevated CSF tau levels are associated with neuronal damage and are also observed in stroke [22] and traumatic brain injury immediately after injury [23], however increases in CSF ptau_181_ levels appear to be specific to AD [24]–[26]. In several previous studies we have successfully applied this endophenotype-based approach, leveraging the information from both CSF Aβ and tau to identify genetic polymorphisms implicated in AD risk [27]–[29].

Tau activity depends on its state of phosphorylation [30], which is regulated by several kinases, phosphatases and other tau-related proteins [31]. Hyperphosphorylation of tau destabilizes the microtubule network, leading to impaired axonal transport and ultimately to neurofibrillary tangle formation and neuronal death (For review see [32]). In the present study we have evaluated 355 single nucleotide polymorphisms (SNPs) in 34 genes involved in tau modification or metabolism for association with CSF levels of ptau_181_, then determined the effects of those variants on AD risk, onset and rate of progression.

## Results

### Association with CSF ptau_181_ levels: Initial screening

**Figure 1:**
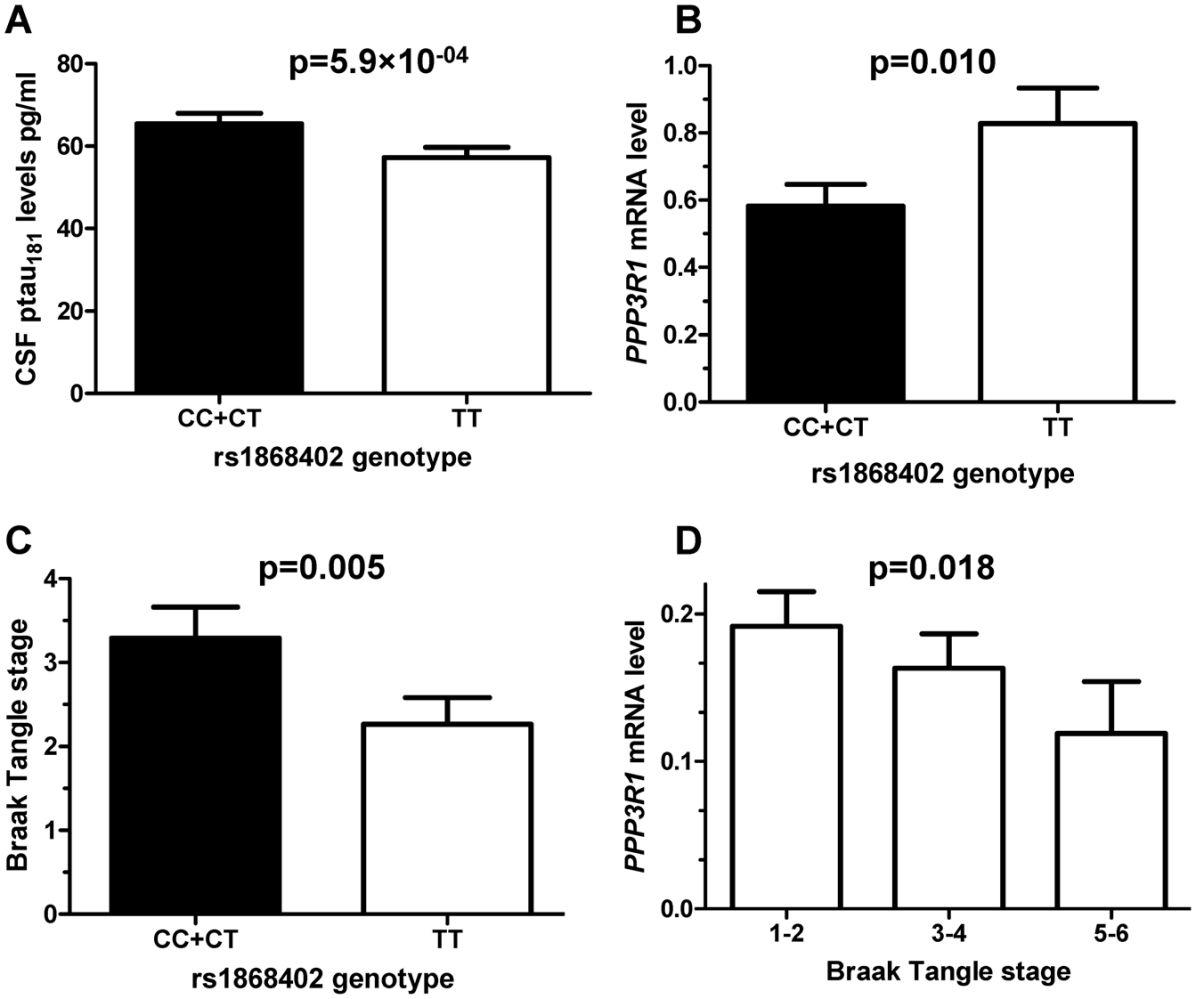
Rs1868402 is associated with CSF ptau_181_ levels, *PPP3R1* mRNA expression levels and tangle counts. **A**. Association of rs1868402 with CSF ptau_181_ levels (WU-ADRC-CSF n = 353) was tested by an Analyses of Covariance (ANCOVA) including CDR, age and *APOE* genotype as covariates. **B**: Minor allele carriers of rs1868402 have significantly lower *PPP3R1* mRNA levels in individuals with AD pathology (n = 82). **C**: Minor allele carriers of rs1868402 have significantly higher numbers of tangles (n = 82). **D**: *PPP3R1* mRNA expression correlates with tangle counts in individuals with AD pathological changes (n = 82). The p-value is for the correlation between mRNA levels and genotypes.

Based on bibliographic data we selected 384 SNPs localized in 34 genes related to tau metabolism (tau kinases, phosphatases, tau O-glcNAcylation or tau degradation (Table S1). 355 SNPs passed quality control (Hardy-Weinberg equilibrium and call rate >95%). Association of SNPs with CSF ptau_181_ levels was evaluated by ANCOVA in 353 CSF samples from the Washington University Alzheimer Disease Research Center (WU-ADRC-CSF) (Tables 1 and 2). Clinical Dementia Rating (CDR), age and *APOE ε4* genotype were included as covariates in the analyses. Eighteen SNPs, located in 7 different genes showed significant association with CSF ptau_181_ levels in the WU-ADRC-CSF series after multiple test correction (Table 3). The SNP with the most significant p-value, rs1868402, is located in intron 5 of the regulatory subunit of the protein phosphatase B gene, also known as calcineurin B (*PPP3R1*; MIM#: 601302). The association of rs1868402 with CSF ptau_181_ levels showed the best fit in the dominant model, with minor alleles carriers showing significantly higher CSF ptau_181_ levels (*P* = 5.90×10^−04^, Figure 1A and Figure S1). All subsequent analyses for rs1868402 used the dominant model. Six other SNPs in *PPP3R1*, which are in high linkage disequilibrium (LD) with rs1868402 (Figure S2, and Table S2), also showed association with CSF ptau_181_. Based on the linkage disequilibrium (LD) in *PPP3R1*, we selected rs1868402 and rs6546366 for replication. The remaining eleven SNPs that were significant after multiple test correction were also selected for replication (Table 3).

**Table 1:** Summary of sample characteristics.

Sample		n	Age (yrs)Mean ± SD (range)	Male (%)	APOEε 4+ (%)	CDR
WU-ADRC-CSF	CSF/Progression	353	68±11 (45–94)	39	40	0 = 72%: >0.5 = 18%
ADNI-CSF	CSF/Progression	236	75±6 (56–91)	56	47	0 = 40%: >0.5 = 60%
UW	CSF	257	69±8 (55–88)	50	50	0 = 52%: >0.5 = 48%
Brain Samples	cases	82	86±7 (72–102)	45	41	>0.5 = 100%
	controls	39	85±9 (64–107)	41	23	0 = 100%
WU-ADRC-CC	cases	340	83±7 (69–101)	35	56	>0.5 = 100%
	control	281	78±8 (60–102)	39	21	0 = 100%
ADNI-CC	cases	100	71±8 (52–91)	55	65	>0.5 = 100%
	control	123	77±5 (61–92)	53	26	0 = 100%
MRC	cases	666	76±7 (60–97)	27	61	>0.5 = 100%
	control	812	76±6 (61–97)	37	23	0 = 100%

Sample size (n), age, percentage of males, percentage of *APOE4* allele carriers, and clinical dementia rating (CDR) for each sample. For the cases age at onset is shown and for controls the age at last assessment.

Washington University Alzheimer's Disease Research Center (ADRC), Alzheimer's Disease Neuroimaging Initiative (ADNI) and for the University of Washington, Seattle (UW). Cerebrospinal Fluid (CSF). Case-control (CC).

**Table 2:** Summary of biomarker characteristics.

	ADRC	ADNI	UW
**Aß_42_**	564±244 (175–1295)	170±56 (53–300)	131±40 (61–231)
**Tau**	376±241 (88–1358)	98±56 (28–495)	72±51 (9–281)
**ptau_181_**	63±32 (24–241)	18±8 (8–115)	63±34 (14–281)

CSF Aß_42_, tau and ptau_181_ levels for the Washington University Alzheimer's Disease Reseach Center (ADRC), Alzheimer's Disease Neuroimaging Initiative (ADNI) and for the University of Washington, Seattle (UW). For each phenotype the mean in pg/ml with the standard deviation and range is shown.

**Table 3:** SNPs associated with CSF ptau_181_ levels in the initial series, the replication series and the combined dataset.

gene	rs	MAF	WU-ADRC-CSF		Replication series		Combined Series	
			uncorrected	FDRp-value	uncorrected	FDRp-value	uncorrected	effect^c^
*PPP3R1*	rs1868402^A^	0.37	**5.90×10^−04^**	**0.025**	**0.005**	**0.034**	1.17×10^−05*^	**−0.30 (0.06)**
*PPP3R1*	rs6546366^A^	0.35	**0.004**	**0.030**	0.271	0.476	0.003	−0.24(0.08)
*F2*	rs2070852^A^	0.32	**0.008**	**0.041**	0.690	0.523	0.114	−0.14 (0.08)
*FYN*	rs927010^B^	0.26	**5.92×10^−04^**	**0.025**	0.425	0.476	0.081	−0.26 (0.17)
*FYN*	rs7768046	0.39	**0.004**	**0.030**	0.465	0.476	0.107	−0.08 (0.06)
*GSK3β*	rs3755557^B^	0.12	**0.004**	**0.030**	0.442	0.476	0.794	0.06 (0.30)
*GSK3β*	rs7431209^A^	0.24	**0.005**	**0.030**	0.946	0.587	0.590	0.03 (0.08)
*MAPT*	rs7210728^A^	0.36	**0.005**	**0.041**	0.529	0.476	0.110	0.11 (0.08)
*MGEA5*	rs2305192^B^	0.30	**0.008**	**0.041**	0.795	0.542	0.046	−0.31 (0.14)
*PRKCA*	rs7218425	0.21	**0.005**	**0.030**	0.358	0.476	0.218	0.07 (0.07)

NPs that passed FDR correction in the WU-ADRC-CSF series were followed up in a replication series composed of CSF samples from ADNI-CSF and UW. Both series (WU-ADRC-CSF and Replication) were combined to increase the statistical power. In the WU-ADRC-CSF and replication series; p-values in bold denote values that are significant after FDR correction. In the Combined Series we applied Bonferroni correction. Threshold for Bonferroni correction is 1.3×10^−04^.

For each SNP the rs number and P values for association with ptau_181_ before and after FDR correction are shown.

A Dominant model.

B Recessive model.

C effects and the standard error of the mean are given in units of standard deviation.

### Association with CSF ptau_181_ levels: Replication in an independent CSF series and combined analyses

In a replication series of 493 independent CSF samples from ADNI (ADNI-CSF) and University of Washington (UW) (Tables 1 and 2) only the SNP located in calcineurin B, rs1868402, replicated and passed the FDR filter (*P* = 0.005 ptau_181_, Table 3, Figure S1). In this series rs1868402 also showed the best fit in the dominant model and minor allele carriers have higher CSF ptau_181_ levels. In the replication CSF series, rs6546366 (*PPP3R1*), showed no association with CSF ptau_181_ levels (Table 3). The lack of association of rs6546366 with CSF ptau_181_ levels is probably due to a lower level of LD with rs1868402 in the replication series (r^2^ = 0.65) compared with the LD between these two SNPs in the WU-ADRC-CSF series (r^2^ = 0.80). This result indicates that rs1868402, or another unknown variant in LD with rs1868402, is the variant that drives the association with CSF ptau_181_ levels.

We also performed a combined analysis by combining the residuals for CSF ptau_181_ after correcting for the covariates (see Materials and Methods). In this analysis we did not included site or platform as a covariate because there were no significant differences between datasets and/or platform for the ptau_181_ residuals. Inclusion of site/platform as a covariate did not significantly change the p-value. In the combined data, rs1868402 showed the most significant association with CSF ptau_181_ levels 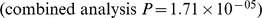, with a p-value that was significant after Bonferroni correction for the entire study 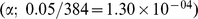. Minor allele carriers have a 2.4 fold increased risk of being in the highest quartile of the CSF ptau_181_ distribution compared to the lowest quartile (odds ratio = 2.37, 95% confidence interval 1.59–3.54). None of the other SNPs were significant after Bonferroni correction (Table 3).

### Association with CSF ptau_181_ levels: Context-dependent effects

It has been demonstrated in our longitudinal data and that of others that the increase in CSF tau and ptau_181_ levels seen in mild AD is preceded by decreases in CSF Aβ_42_ levels [14], [21]. This likely reflects deposition of Aβ in the brain [14]. Individuals with CSF Aβ_42_ levels less than 500 pg/ml in the WU-ADRC-CSF, and less than 192 pg/ml in the ADNI-CSF series, have evidence of Aβ deposition in the brain, as detected by PET-PIB [14], [21]. We used these CSF Aβ_42_ thresholds to stratify the WU-ADRC-CSF and ADNI-CSF samples into individuals with low or high CSF Aβ_42_ levels (with and without likely Aβ deposition in the brain). The difference in CSF Aβ_42_ threshold levels between the WU-ADRC-CSF and ADNI-CSF series is due to different antibodies and procedures used to measure the CSF levels (see Materials and Methods). The data necessary to examine the correlation between the CSF Aβ_42_ levels and PET-PIB signal is not available in the UW CSF series and therefore this dataset could not be included in this analysis. In these analyses we calculated the p-value and the Odds ratios for rs1868402 with CSF ptau_181_ levels by comparing the frequency of this SNP in the lowest versus the highest quartile of the CSF ptau_181_ levels after correcting for the covariates. When we stratified the WU-ADRC-CSF and ADNI-CSF series by CSF Aβ_42_ levels, we observed very significant association for rs1868402 with CSF ptau_181_ in the low Aβ_42_ stratum (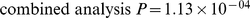, Odds Ratio 3.48; 95% confidence interval 1.8–6.7) and a nominally significant association in the high Aβ_42_ stratum (combined analysis *P* = 0.023; Odds Ratio 2.54; 95% confidence interval 1.0–6.75, Table 4)._._ Both the high and low Aβ_42_ level strata have sufficient power to detect the association between rs1868402 with CSF ptau_181_ levels (high Aβ_42_ 0.985; low Aβ_42_ = 0.975; α = 0.05). The nominally significant p-value in the high Aβ stratum may indicate a moderate effect on CSF ptau_181_ levels in healthy individuals, but it is clear that when AD pathology is present the effect of this SNP is more marked.

**Table 4:** Rs 1868402 is associated with CSF ptau_181_ levels in individuals with Aβ deposition.

Gene	Rs #	stratum	n	ptau_181_	Effect^B^	OR (95%)
*PPP3R1*	rs1868402^A^	Total sample	589	**2.62×10^−05^**	**−0.34 (0.08)**	2.78 (1.73–4.54)
		Low Aβ levels	300	**1.13×10^−04^**	**−0.47 (0.12)**	3.48 (1.80–6.75)
		High Aβ levels	289	0.023	**−0.18 (0.10)**	2.54 (1.00–5.75)

The p-value and the OR for rs1868402 were calculated by comparing the rs1868402 allele frequency in the lowest quartile vs the highest quartile of CSF ptau_181_ levels, after correction for covariates in the WU-ADRC-CSF (n = 353)+ADNI-CSF (n = 266) samples. CSF Samples from the UW could not be included in this analysis because there is no study analyzing the correlation between the CSF Aβ_42_ levels and PET-PIB signal in this dataset, and therefore the threshold of CSF Aβ_42_ levels for PIB positivity is unknown. OR (95%) = Odds ratio with the 95% confidence interval: Odds ratios were calculated comparing the highest vs lowest quartile of CSF ptau_181_ levels. Samples were stratified based on CSF Aβ_42_ levels as an approximation of Aβ deposition. For the ADRC samples individuals with Aβ_42_ levels less than 500 pg/ml were considered positive for Aβ deposition and for ADNI samples the Aβ_42_ cutoff value of 192 pg/ml was used.

Values in boldface indicate significant p-values.

A Dominant model.

B effects and the standard error of the mean are given in units of standard deviation.

Rs1868402 explains 4.62% of the variability in CSF ptau_181_ levels in individuals with low CSF Aβ_42_ levels in the WU-ADRC-CSF+ADNI-CSF samples, which is similar to the variability explained by other SNPs and endophenotypes [3]–[11]. It is important to note that in the low CSF Aβ_42_ group there are individuals diagnosed with DAT (CDR>0, n = 183, 58%) and non-demented individuals (CDR = 0, n = 134, 42%) with possible Aβ deposition in the brain and brain atrophy (presymptomatic AD) [12]. In the high Aβ_42_ stratum 80% of the samples (n = 242) had a CDR = 0.

### Implication of SNPs associated with CSF ptau_181_ levels in AD: Association with rate of progression of AD but not risk for AD or age at onset

**Figure 2:**
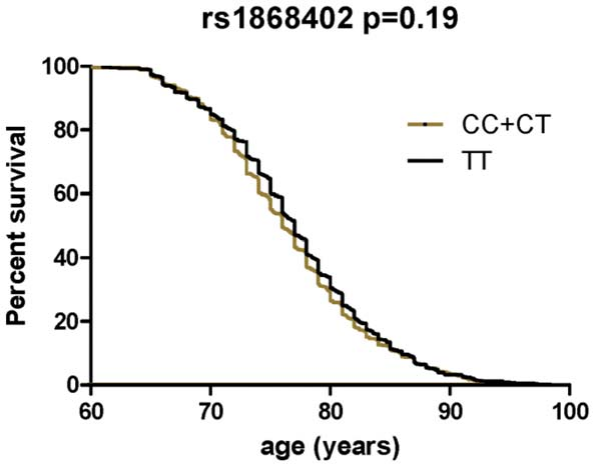
Survival curves comparing age at onset of LOAD between the different genotypes of rs1868402. Survival fractions were calculated using the Kaplan-Meier method and significant differences were calculated by Log-rank test. Association with age at onset was calculated in a combined series with samples from WU-ADRC-CC, ADNI-CC and MRC.

The premise of this endophenotype-based approach is that a SNP, such as rs1868402 that shows strong, replicable association with an important AD biomarker should also modulate risk, onset and/or progression of AD. We tested whether rs1868402 influences risk for AD, age at onset and disease progression. We found no association between rs1868402 and risk for AD (*P* = 0.10, Table 5) or age at onset (*P* = 0.19 Figure 2) in 1106 cases and 1216 controls of European descent.

**Table 5:** Rs1868402 is not associated with risk for AD.

					MAF		
	Series	Cases	Controls	Minor Allele	Cases	Controls	p-value
**rs1868402**	MRC	666	812	C	0.512	0.471	0.06
	WU ADRC-CC	340	281	C	0.497	0.470	0.25
	ADNI-CC	100	123	C	0.505	0.446	0.11
	**Total**	**1106**	**1216**	C	0.504	0.467	0.10

rs1868402 was genotyped in the MRC, WU-ADRC-CC and ADNI-CC series. Number of cases and controls, minor allele and minor allele frequency (MAF) for each series and for the combined series are showed. P-value for the dominant (rs1868402) model were calculated by logistic regression including *APOE*, age, gender and series as covariates.

To examine disease progression we used two longitudinal datasets: 109 subjects from WU-ADRC-CSF (399 observations) and 150 subjects from ADNI-CSF (620 observations). Association with rate of progression was evaluated by comparing the change in sum of boxes of the CDR (SB-CDR) per year (slope) by genotype including age at the first visit, gender, *APOE* genotype and initial CDR as covariates. CSF ptau_181_ and Aβ_42_ levels were also included in the model to correct for the potential association between these phenotypes with progression [33]. Because the association of rs1868402 with CSF ptau_181_ was mainly in individuals with low CSF Aβ_42_ levels, association with progression was analyzed in individuals with low CSF Aβ_42_ levels (less than 500 pg/ml in the WU-ADRC-CSF, and less than 192 pg/ml in the ADNI-CSF series), and an initial CDR of 0 or 0.5. Individuals with CDR 0 and 0.5 were selected to maximize the amount of information on progression per individual and to avoid possible ceiling effects from individuals who began the study with advanced levels of dementia.

**Figure 3:**
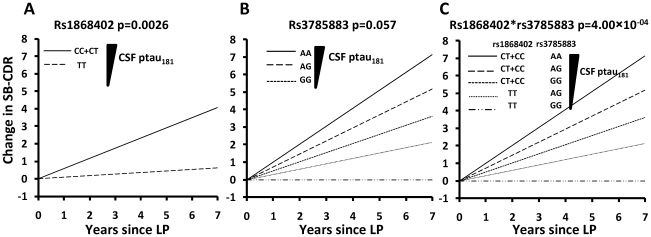
Genetic variants associated with CSF ptau_181_ levels are also associated with rate of progression of AD. Rate of progression is defined as the change in the Clinical Dementia Rating sum of boxes (SB-CDR) score per year. Association of SNPs with progression was calculated using a mixed linear model (PROC MIXED) after controlling for age, sex, *APOE*, *initial CDR*, CSF ptau_181_ and Aβ_42_ levels. **A**. Minor allele carriers of rs1868402, are associated with higher CSF ptau_181_ levels, and show a 6 fold faster progression than homozygotes for the major allele (CDR-SB/year: 0.58 vs 0.09; p = 0.0026) in individuals from the WU-ADRC-CSF with low CSF Aβ_42_ levels (<500pg/ml). For this SNP the dominant model was used because it showed the best fit in all the analyses. **B**. rs3785883 genotypes do not have significantly different progression rates *P* = 0.057. The genotype frequency distribution for rs3785883 with disease progression is most likely not significant due to the low statistical power. AA carriers show a CDR-SB of 1.01, AG of 0.47 and GG 0.26 (p = 0.057). The additive model was used because it showed the best fit. **C**. Rs1868402 and rs3785883 show an epistatic interaction. Carriers of the alleles associated with higher CSF ptau_181_ levels (CT+CC for rs1868402 and AA for 3785883) showed a CDR-SB/year of 1.02 vs −0.006 for carriers of alleles associated with lowest CSF ptau_181_ levels. LP indicates lumbar puncture.

Carriers of the rs1868402 allele associated with higher CSF ptau_181_ levels showed an increase of 0.58 SB-CDR per year, which is six-fold faster than the rate seen in individuals homozygous for the allele associated with low CSF ptau_181_ levels (*P* = 0.0026; Table 6, Figure 3A and Figure S3), and almost two times faster than the average change for the entire series (SB-CDR per year for the entire series 0.31). The association of rs1868402 with progression replicated in the ADNI-CSF series (*P* = 0.014) with a *P*_combined_ = 1.96×10^−05^. In addition, we also used the ADNI samples with no CSF data (ADNI-CC, Table 6) to replicate the association with rate of progression in an independent sample. In this dataset rs1868402 also showed a significant association with rate of progression (*P* = 0.018, Table 6) and in the same direction as in the previous analyses.

**Table 6:** Association with rate of disease progression.

	WU-ADRC-CSF	ADNI-CSF	Combined CSF series	ADNI samplesNo CSF
**rs1868402**	0.0026	0.014	1.71×10^−05^	0.0187
rs1868402*rs3785883	4.00×10^−04^	0.010	1.43×10^−05^	0.0123

Rs1868402 is associated with progression rate and shows epistasis with rs3785883, which is also associated with CSF tau levels [27].

Rate of progression were analyzed by the change of the Clinical Dementia Rating sum of boxes (CDR-SB) score per year in individuals with low CSF Aβ_42_ levels (ADRC<500pg/ml; ADNI<192pg/ml) and in samples with no CSF data. Association of SNPs with progression was calculated with mixed linear models (proc mixed) after controlling for age, sex, *APOE*, CDR and/or CSF ptau_181_ and Aβ_42_.

We tested whether rs1868402 interacts with rs3785883, a SNP located in *MAPT*, which is also associated with CSF ptau_181_ levels [27]. Rs3785883 also showed a p-value for association with CSF ptau_181_ levels of 0.008 in the combined series of this study.We found significant epistasis between these SNPs. Individuals carrying alleles associated with higher CSF ptau_181_ for rs1868402 and rs3785883 showed an increase of 1.02 SB-CDR per year on average, whereas those carrying the alleles associated with lower CSF ptau_181_ at each SNP showed essentially no change in the SB-CDR per year (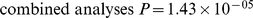; Figure 3B; Table 6). The interaction between rs1868402 and rs3785883 also replicated in the ADNI-CSF and ADNI-CC series (Table 6).

### Gene expression

Since rs1868402 is located in a region that encodes a tau phosphatase, we tested next whether the SNP is associated with *PPP3R1* mRNA levels and tau pathology in brain. We extracted total RNA from the parietal lobe of 82 AD cases and 39 non-demented elderly individuals. The allele of rs1868402, associated with higher CSF ptau_181_ levels, showed significantly lower *PPP3R1* mRNA levels (*P* = 0.010; Figure 1B), and higher tangle pathology as measured by Braak stage (*P* = 0.005; Figure 1C) in brain samples with Aβ pathology, but not in neuropathologically normal samples. We also found that there was a correlation between *PPP3R1* mRNA levels and Braak stage in these samples (*P* = 0.018; Figure 1D). Rs12713636, in LD with rs1868402 (D′ = 1, R^2^ = 0.75) also shows association with *PPP3R1* mRNA levels and in the same direction in the publicly available GEO GSE8919 [34] dataset (*P* = 0.015).

## Discussion

In the present study we have used a novel and powerful endophenotype-based approach to identify a novel genetic factor implicated in AD. Most genetic studies in AD have focused on identifying genetic factors that modulate risk or age at onset of disease [1]–[2]. The genetic factors influence other important facets of AD, such as rate of progression or disease duration remain poorly understood. Here we report that the minor allele of rs1868402 shows significant, replicable association with higher CSF ptau_181_ levels and faster rate of progression of AD. We failed to detect evidence for association with risk for disease or age at onset. This is consistent with the known pathobiology of AD, in which Aβ aggregation and deposition is an early preclinical event, followed by increased CSF tau and ptau_181_ levels and tau pathology during the clinical phase of the disease. Under this model, factors that affect tau phosphorylation and aggregation may be expected to modify disease progression but not risk for disease, while genes that influence Aβ aggregation, such as *APOE4* [35], would be expected to influence CSF Aβ_42_ levels and risk/age at onset of disease but not CSF tau/ptau_181_ levels or rate of progression of disease. Table 7 illustrates that this is the case in our data. Genetic analyses of CSF Aβ_42_ and tau variation therefore allow identification of genetic factors that influence different components of the disease process.

**Table 7:** Variants that modify CSF Aβ_42_ levels affect risk for AD, whereas variants associated with CSF ptau_181_ levels affect rate of progression.

Association with	CSF ptau_181_ *	CSF Aβ_42_ *	Risk**	age at onset**	Progression***
**rs1868402**	**1.17×10^−05^** *	0.53	0.31	0.47	**1.31×10^−04^**
***APOE4***	0.018	**3.9×10^−9^** *	**3.9×10^−40^**	**4.0×10^−7^**	0.136

P-values for the association of rs1868402 (*PPP3R1*) and *APOE4* with CSF Aß_42_ and ptau_181_ levels, risk for AD, age at onset and disease progression are shown.

*Sample size = 846; WU-ADRC-CSF+ADNI-CSF+UW.

**Sample size = 2322 MRC+WU ADRC-CC+ADNI-CC.

***Sample size** = 259**; WU-ADRC-CSF+ADNI-CSF with low CSF Aβ_42_ levels.

Significant p-values are in bold.

Our results suggest that rs1868402, or another variant in LD with it, may reduce calcineurin expression/activity leading to an increase in tau phosphorylation increasing tau pathology and neurodegeneration in individuals with Aβ deposition. The resulting increase in tau-related pathology would then increase the rate of progression of AD. Several studies provide support for a role of calcineurin in AD pathogenesis. Inhibition of calcineurin in mouse brains by cyclosporin A or FK506 or rat brain by antisense oligonucleotides led to enhanced tau phosphorylation [36]–[38]. In mice the increase in tau phosphorylation was accompanied by impaired spatial memory, a characteristic feature of AD [36]. Finally, in AD patients, calcineurin activity is decreased and correlates with neuropathologic changes [39].

Our analyses suggest that genetic variants associated with CSF Aβ_42_ levels also influence risk and age at onset (e.g. *APOE*) but variants associated with CSF ptau_181_ levels have a greater impact on rate of progression (Table 7). Genome-wide association studies of CSF tau/ptau_181_ levels should identify novel genetic variants which will likely influence rate of progression of AD. Variants that influence disease progression may have significant clinical benefit. For example, these variants have the potential to predict more accurately the time from diagnosis to functional impairment that may require nursing home placement. Stratification of samples by such SNPs will enable cheaper and more efficient clinical trials by selecting individuals expected to have faster rates of progression. By targeting different facets of AD biology this approach can identify a broader range of potential therapeutic targets than a conventional case-control design. Drugs that inhibit or decrease tau phosphorylation would be expected to decrease cognitive decline in individuals with very mild dementia or delay the appearance of memory problems in elderly individuals with low CSF Aβ_42_ levels. Finally, we believe that this approach is applicable to other common neurological and psychiatric disorders, where biomarkers of disease have been identified, and underlines the value and importance of finding such markers in other diseases.

## Materials and Methods

### Subjects and endophenotypes

The cerebrospinal fluid discovery series includes 353 individuals enrolled in longitudinal studies at the WU-ADRC. CSF collection and Aβ_42_, tau and ptau_181_ measurements were performed as described previously [14]. Table 1 shows the demographic data for the CSF series and Table 2 shows a description of the CSF biomarker in each dataset. The CSF replication series consists of 236 individuals from the ADNI dataset and 257 individuals from the University of Washington (UW, Seattle). All CSF samples were from individuals of European descent. Written consent was obtained from all participants. While there are differences in the absolute levels of the biomarker measurements between the two studies that likely reflect differences in the methods used for quantification (regular ELISA vs Luminex), ascertainment, and/or in handling of the CSF after collection, CSF ptau_181_ levels in the WU-ADRC-CSF, ADNI-CSF, and UW samples show similar characteristics. CSF ptau_181_ levels show a 10–17 fold difference between individuals in each dataset, are normally distributed after log-log transformation, and have similar covariates in each dataset (see statistical analyses).

Risk for disease and age at onset analyses were analyzed in a total of 1106 late-onset AD (LOAD) cases and 1216 age-gender-ethnicity matched non-demented controls (Table 5). These samples were ascertained at the WU-ADRC, MRC genetic resource for late-onset AD (UK, MRC Sample [40]), and ADNI. Cases received a diagnosis of dementia of the Alzheimer's type (DAT), using criteria equivalent to the National Institute of Neurological and Communication Disorders and Stroke-Alzheimer's Disease and Related Disorders Association for probable AD [41]–[42]. All individuals were of European descent and written consent was obtained from all participants.

Association of rate of progression of dementia with genetic variants was tested in two longitudinal series from the WU-ADRC and ADNI. The WU-ADRC-CSF series includes 109 individuals with clinical data from at least two time points starting with a CDR of 0 or 0.5 in the first interview, and a diagnosis of DAT (dementia Alzheimer Type) at the last visit. There are an average of 3.8 observations per individual, which varies from 2 to 14 with an average follow up time of 3.2 years. The second series with longitudinal data is the ADNI series: 459 individuals (236 with CSF data and another 223 with no CSF data) have had a clinical examination at least two time points with an average of 4.1 observations per individual, which varies between two and six observations, however the average follow up time is only 1.9 years. To study the association with progression rate in the CSF samples we analyze only the 150 samples with low (<192pg/ml) CSF Aβ_42_, as explained in the Statistical Analyses section.

### SNP selection and genotyping

Based on bibliographic data, we selected 384 SNPs in the most relevant tau kinases, phosphatases, and in other genes implicated in other posttranslational modifications of tau, or tau degradation [45]–[47] (Table S1). Tagging SNPs (r^2^>0.8), based on CEU-HapMap data, were selected for each of these genes. We used Pupasuite software [48] to select potentially functional variants in the selected genes and flanking regions. SNPs were genotyped using the Illumina Golden Gate, Sequenom and/or Taqman genotyping technologies. Only SNPs with a genotyping call rate higher than 95% and in Hardy-Weinberg equilibrium were used in the analyses.

### Gene expression

Expression studies were carried out using cDNA obtained from the parietal lobe of 82 AD cases and 39 non-demented individuals (CDR = 0) obtained through the WU-ADRC Neuropathology Core (Brain samples; Table 1). AD changes were measured using Braak and Braak staging [43]. All AD cases had a Braak and Braak score of 5 or 6. Among the non-demented individuals 24 brains had a Braak and Braak score ranging from 1–4 indicating the presence of some tangle pathology.

Total RNA was extracted from the parietal lobe of 82 AD cases and 39 non-demented individuals, using the RNeasy mini kit (Qiagen) following the manufacturer's protocol. cDNAs were prepared from the total RNA, using the High-Capacity cDNA Archive kit (ABI). Gene expression was analyzed by real-time PCR, using an ABI-7500 real-time PCR system. Real-time PCR assays were used to quantify *PPP3R1* cDNA levels. Taqman assays for *GAPDH* (sequences available on request) *PPP3R1* (ABI: C_12044272_10) and cyclophilin A (ABI: 4326316E) were used to quantify the gene expression levels. Each real-time PCR run included within-plate duplicates and each experiment was performed, at least twice for each sample. Real-time data were analyzed using the comparative Ct method. The Ct values of each sample were normalized with the Ct value for the housekeeping genes, GADPH and cyclophilin, and were corrected for the PCR efficiency of each assay [44], although the efficiency of all reactions was close to 100%. Only samples with a standard error of <0.15% were analyzed.

### Statistical analyses

CSF ptau_181_ values were log-log transformed to approximate a normal distribution. Analysis of the covariance (ANCOVA) was used to test for association between genotypes and CSF ptau_181_ levels. In order to identify the covariates that affect CSF ptau_181_ levels, we performed a stepwise discriminant analysis including CDR, age, gender and *APOE* genotype. CDR, age, and *APOE* genotype were identified as significant covariates in the WU-ADRC-CSF series and, CDR and *APOE* genotype in the replication series (this series has a narrower age range than the WU-ADRC-CSF series). These covariates were included in the respective ANCOVA. Each SNP was tested using an additive model with minor allele homozygotes coded as 0, heterozygotes coded as 1, and major allele homozygotes coded as 2. When the additive model was significant after multiple test correction, dominant and recessive models were tested to determine whether the y were a better fit. Because the CSF ptau_181_ levels in the WU-ADRC-CSF, ADNI-CSF and UW samples were measured using different platforms (Innotest plate ELISA vs AlzBia3 bead-based ELISA, respectively) we were not able to combine the raw data, rather we combined the residual values of the CSF ptau_181_ obtained after correcting for the covariates. No significant differences in the residuals from the different series were found, indicating that the differences in the CSF levels due to the different platforms were corrected by using the residuals.

Multiple test correction: Initial tests for association of SNPs with CSF ptau_181_ levels were evaluated using a False Discovery Rate (FDR) filter of 0.1 to correct for multiple testing [49]. In the initial screening only p-values more significant than 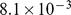 passed FDR filter of 0.1. In the replication series p-values more significant than 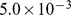 passed FDR filter. To reduce the probability of false positives we also used the more stringent Bonferroni correction to adjust the alpha level in the analysis of association with CSF ptau_181_ levels in the combined samples. In this case, the threshold for Bonferroni correction for the combined sample is 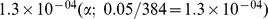. No multiple test correction was applied for association with risk for disease, age at onset or progression because only one or two SNPs with specific hypotheses were tested for association. To calculate the impact of rs1868402 on the CSF ptau_181_ levels, we calculated the Odds Ratio (OR) of this SNP by comparing its frequency in the highest vs lowest quartile of the residuals for CSF ptau_181_ levels.

Allelic association with risk for AD was tested using logistic regression including *APOE*, gender, age and series as covariates. Association with AAO was carried out using the Kaplan-Meier method and tested for significant differences, using a log-rank test. Association with rate of disease progression was evaluated as described previously [33]. Briefly, progression of disease was measured by the change in sum of boxes on the CDR (clinical dementia rate; SB-CDR) per year. CDR is a global measurement of the severity of symptoms of dementia. CDR evaluates cognitive and functional performance in six areas (memory, orientation, judgment and problem solving, community affairs, home and hobbies and, personal care), each of these areas has a possible score of 0, 0.5, 1, 2 or 3. The sum of boxes can vary between 0 and 18. Higher scores indicate more significant memory problems and correlate with neurodegeneration [50]. The change in SB-CDR per year fitted a linear model in both series and therefore we used a mixed linear model (PROC MIXED; SAS Institute Inc) to determine whether there is a relationship between the slope of the SB-CDR score and time as a function of genotype after controlling for initial age, gender, *APOE*, initial CDR, CSF ptau_181_ and Aβ_42_ levels. Because the association between rs1868402 and CSF ptau_181_ was driven by individuals with low CSF Aβ_42_ levels, association with progression in the CSF datasets was analyzed only in individuals with low CSF Aβ_42_ levels (less than 500 pg/ml in the WU-ADRC-CSF series [14] or 192 pg/ml in the ADNI-CSF series [21]). We analyzed whether the combination of genotypes of rs1868402 and rs3785883 predicts the rate of progression better than either of these SNPs alone. To do that we included an interaction term rs1868402*rs3785883 in the “proc mixed” SAS program, that combine the genotypes for the two SNPs. We also combined the data from the WU-ADRC-CSF and ADNI-CSF to increase the statistical power.

Association between cDNA levels, tau pathology (Braak tangle stage) and genotypes were carried out using ANCOVA. Stepwise discriminant analysis was used to determine the significant covariates (age, gender, postmortem interval, *APOE* genotype, and CDR). One-tailed P-values were calculated, because *a priori* predictions were made based on the associations with CSF ptau_181_ levels. We also used the GEO dataset GSE8919 [51] to analyze the association between rs12713636, in LD with rs1868402 (D′ = 1, R^2^ = 0.75) and *PPP3R1* gene expression. In this dataset there are genotype and expression data from 486 Late onset Alzheimer Diseases cases and 279 neuropathologically confirmed controls. We only extracted the normalized *PPP3R1* mRNA levels and the genotype data for rs12713636. Genotypes for rs12713636 were used because rs1868402 was not included in this dataset.

### ADNI material and methods

Data used in the preparation of this article were obtained from the ADNI database (www.loni.ucla.edu/ADNI). The ADNI was launched in 2003 by the National Institute on Aging, the National Institute of Biomedical Imaging and Bioengineering, the Food and Drug Administration, private pharmaceutical companies and non-profit organizations, as a $60 million, 5-year public-private partnership. The Principal Investigator of this initiative is Michael W. Weiner, M.D. ADNI is the result of efforts of many co-investigators from a broad range of academic institutions and private corporations, and subjects have been recruited from over 50 sites across the U.S. and Canada. The initial goal of ADNI was to recruit 800 adults, ages 55 to 90, to participate in the research -approximately 200 cognitively normal older individuals to be followed for 3 years, 400 people with MCI to be followed for 3 years, and 200 people with early AD to be followed for 2 years.” For up-to-date information see www.adni-info.org.

## Supporting Information

Figure S1 Minor allele carriers of rs1868402 present significantly higher CSF ptau_181_ levels. The mean and the standard error of the mean (SEM) for the raw and residuals CSF ptau_181_ levels for the WU-ADRC-CSF, ADNI-CSF and UW series is shown. A. Raw CSF ptau_181_ levels for the WU-ADRC-CSF series by rs1868402 genotype. CC+CT: 64.45±2.52. TT: 57.20±2.49 pg/ml. B. Raw CSF ptau_181_ levels for the ADNI-CSF series by rs1868402 genotype. CC+CT: 35.95±1.85. TT: 30.22±1.44 pg/ml. C. Raw CSF ptau_181_ levels for the UW series by rs1868402 genotype. CC+CT: 67.46±3.53. TT: 61.42±2.71 pg/ml. D. Residuals CSF ptau_181_ levels for the WU-ADRC-CSF series by rs1868402 genotype. CC+CT: 0.17±0.07. TT: −0.22±0.08. E. Residuals CSF ptau_181_ levels for the ADNI-CSF series by rs1868402 genotype. CC+CT: 0.13±0.09. TT: −0.16±0.08. F. Residuals CSF ptau_181_ levels for the UW series by rs1868402 genotype. CC+CT: 0.10±0.08. TT: −0.12±0.09.
(0.06 MB DOC)

Figure S2 Linkage disequilibrium among *PPP3R1* SNPs significantly associated with CSF tau levels in the ADRC series. Color represents D′ = 1 and numbers correspond to r2.
(0.16 MB DOC)

Figure S3 Genetic variants associated with CSF ptau_181_ levels are also associated with rate of progression. Average progression rate by genotype with the 95% confidence interval. Solid lines represent the average progression rate. Dotted lines represent the 95% confidence interval. The lines for the different genotypes are color code. A. Minor allele carriers of rs1868402, are associated with higher CSF ptau_181_ levels, and show a 6-fold faster progression than homozygotes for the major allele (CDR-SB/year: 0.58 vs. 0.09; p = 0.0026) in individuals from the WU-ADRC-CSF with low CSF Aβ_42_ levels (<500pg/ml). B. rs3785883 genotypes do not have significantly different progression rates P = 0.057. The genotype frequency distribution for rs3785883 with disease progression is most likely not significant due to the low statistical power. AA carriers show a CDR-SB of 1.01, AG of 0.47 and GG 0.26 (p = 0.057).
(0.23 MB DOC)

Table S1 Genes and SNPs/gene genotyped in this study. The official and the most common alias of the gene, the activity related to tau, chromosomal position, and gene size in Kb are shown. A. Tag SNP. SNPs that capture 80% of the common sequence diversity within the gene. B. Only validated SNP with a minor allele frequency >0.1. C. Number of SNPs that passed quality controls.
(0.09 MB DOC)

Table S2 SNPs in *PPP3R1* associated with CSF ptau_181_ levels in the discovery series (WU-ADRC-CSF). SNPs associated with CSF ptau_181_ levels after FDR correction are shown. MAF = Minor Allele Frequency. A: Dominant model. B: Recessive model.
(0.03 MB DOC)
