# Supplementary material for: SNPs Associated with Cerebrospinal Fluid Phospho-Tau Levels Influence Rate of Decline in Alzheimer's Disease
Source: PLoS Genet. 2010 Sep 16;6(9):e1001101. doi: 10.1371/journal.pgen.1001101 (PMC2940763; doi:10.1371/journal.pgen.1001101)
Supplement: Figure S1 — Minor allele carriers of rs1868402 present significantly higher CSF ptau181 levels. The mean and the standard error of the mean (SEM) for the raw and residuals CSF ptau181 levels for the WU-ADRC-CSF, ADNI-CSF and UW series is shown. A. Raw CSF ptau181 levels for the WU-ADRC-CSF series by rs1868402 genotype. CC+CT: 64.45±2.52. TT: 57.20±2.49 pg/ml. B. Raw CSF ptau181 levels for the ADNI-CSF series by rs1868402 genotype. CC+CT: 35.95±1.85. TT: 30.22±1.44 pg/ml. C. Raw CSF ptau181 levels for the UW series by rs1868402 genotype. CC+CT: 67.46±3.53. TT: 61.42±2.71 pg/ml. D. Residuals CSF ptau181 levels for the WU-ADRC-CSF series by rs1868402 genotype. CC+CT: 0.17±0.07. TT: −0.22±0.08. E. Residuals CSF ptau181 levels for the ADNI-CSF series by rs1868402 genotype. CC+CT: 0.13±0.09. TT: −0.16±0.08. F. Residuals CSF ptau181 levels for the UW series by rs1868402 genotype. CC+CT: 0.10±0.08. TT: −0.12±0.09. (0.06 MB DOC) [file pgen.1001101.s001.doc]

**Supplementary Figure 1:**

**A**

**B**

**C**

**D**

**E**

**F**
