# Supplementary figures and images for: SNPs Associated with Cerebrospinal Fluid Phospho-Tau Levels Influence Rate of Decline in Alzheimer's Disease
Source: PLoS Genet. 2010 Sep 16;6(9):e1001101. doi: 10.1371/journal.pgen.1001101 (PMC2940763; doi:10.1371/journal.pgen.1001101)

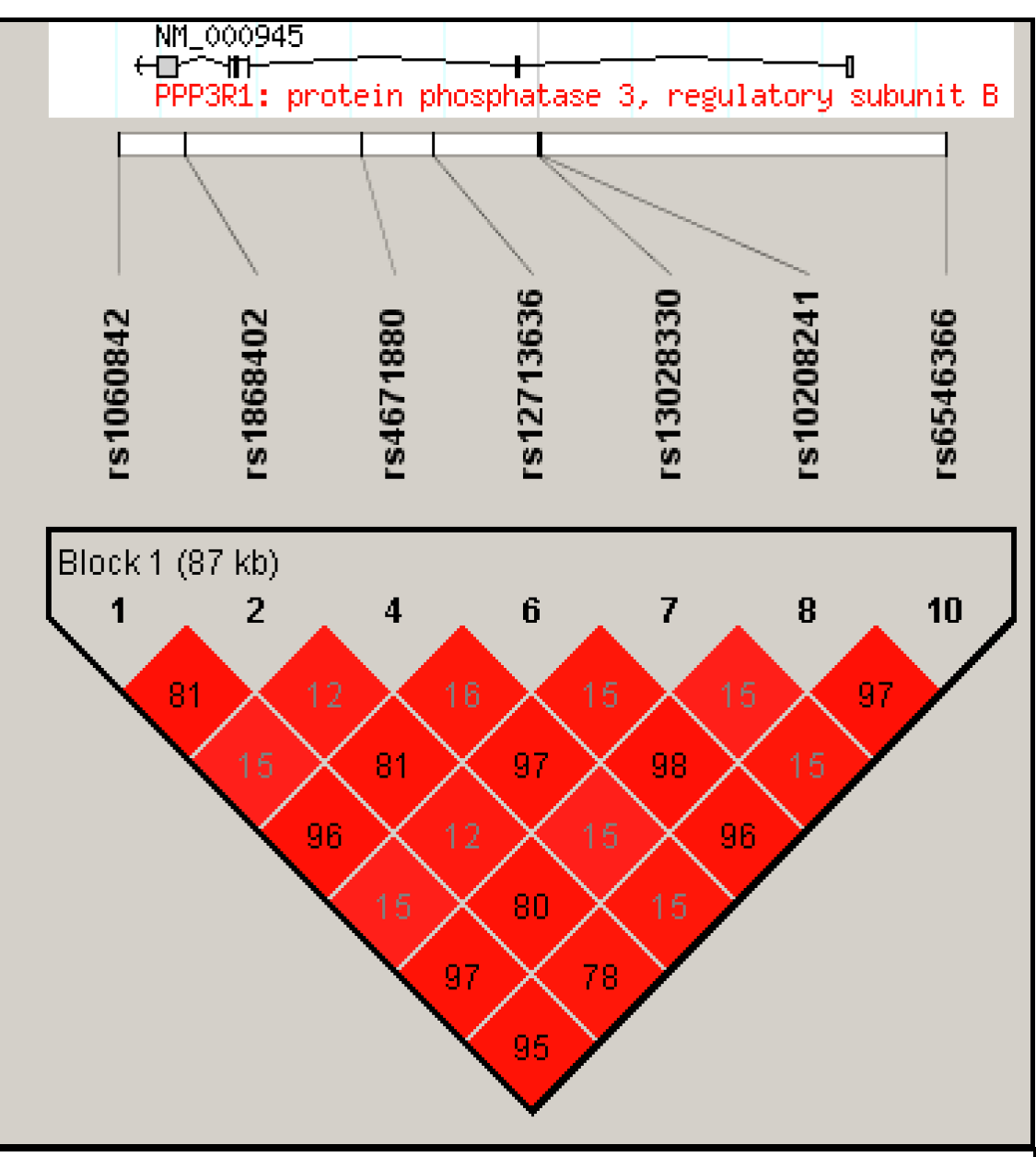

Supplement: Figure S2 — Linkage disequilibrium among PPP3R1 SNPs significantly associated with CSF tau levels in the ADRC series. Color represents D′ = 1 and numbers correspond to r2. (0.16 MB DOC) [file pgen.1001101.s002.doc]

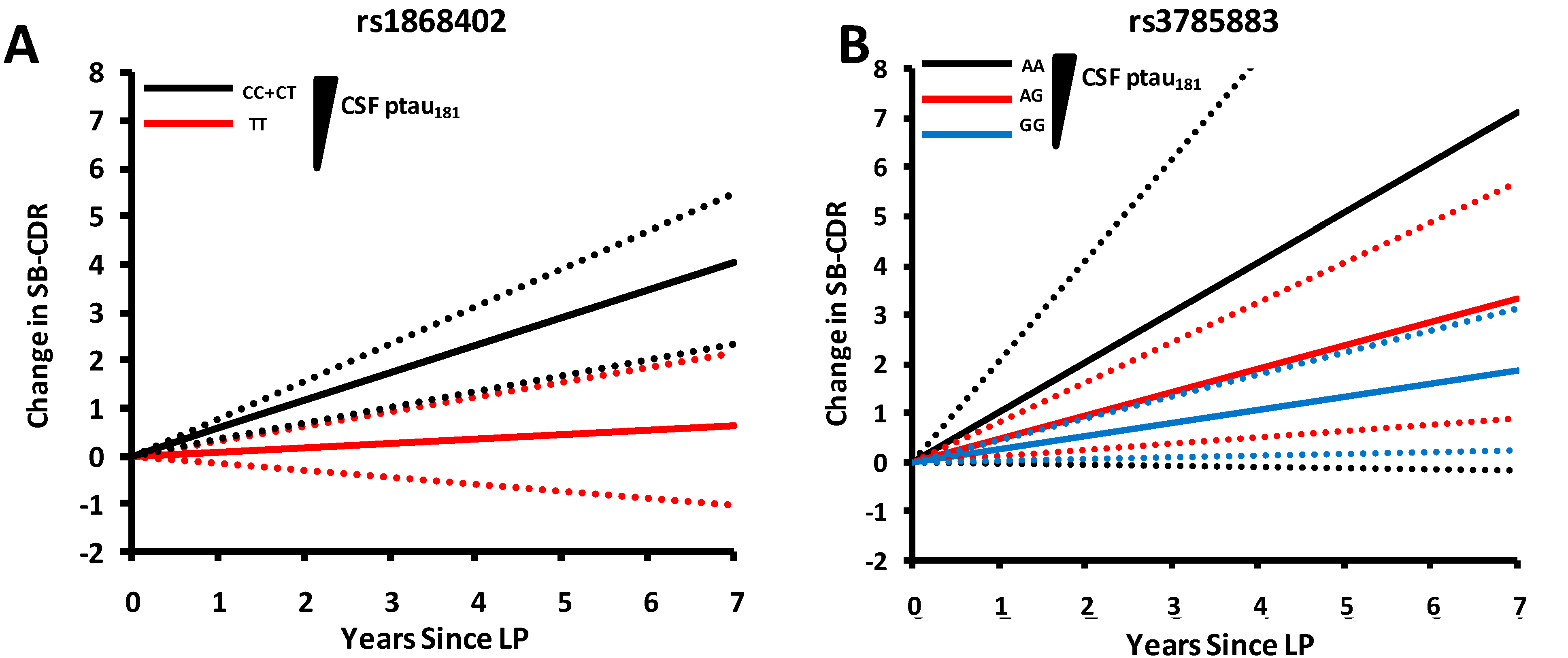

Supplement: Figure S3 — Genetic variants associated with CSF ptau181 levels are also associated with rate of progression. Average progression rate by genotype with the 95% confidence interval. Solid lines represent the average progression rate. Dotted lines represent the 95% confidence interval. The lines for the different genotypes are color code. A. Minor allele carriers of rs1868402, are associated with higher CSF ptau181 levels, and show a 6-fold faster progression than homozygotes for the major allele (CDR-SB/year: 0.58 vs. 0.09; p = 0.0026) in individuals from the WU-ADRC-CSF with low CSF Aβ42 levels (<500pg/ml). B. rs3785883 genotypes do not have significantly different progression rates P = 0.057. The genotype frequency distribution for rs3785883 with disease progression is most likely not significant due to the low statistical power. AA carriers show a CDR-SB of 1.01, AG of 0.47 and GG 0.26 (p = 0.057). (0.23 MB DOC) [file pgen.1001101.s003.doc]
