# Supplementary material for: SNPs Associated with Cerebrospinal Fluid Phospho-Tau Levels Influence Rate of Decline in Alzheimer's Disease
Source: PLoS Genet. 2010 Sep 16;6(9):e1001101. doi: 10.1371/journal.pgen.1001101 (PMC2940763; doi:10.1371/journal.pgen.1001101)
Supplement: Table S1 — Genes and SNPs/gene genotyped in this study. The official and the most common alias of the gene, the activity related to tau, chromosomal position, and gene size in Kb are shown. A. Tag SNP. SNPs that capture 80% of the common sequence diversity within the gene. B. Only validated SNP with a minor allele frequency >0.1. C. Number of SNPs that passed quality controls. (0.09 MB DOC) [file pgen.1001101.s004.doc]

|  | **Official Name** | **Common Alias** | **Activity** | **Chr.** | **Size Kb** | **tSNP^A^** | **Evolutionary Conserved^B^** | **Potentially functional^B^** | **Total**  **Selected** | **Passed**  **QC^C^** |
| --- | --- | --- | --- | --- | --- | --- | --- | --- | --- | --- |
| 1 | MARK1 | - | Phosphorylation | 1q41 | 135.7 | 7 | 4 | 1 | 12 | 8 |
| 2 | PPP3R1 | CNB | Phosphorylation | 2p15 | 73.65 | 8 | 2 | 1 | 11 | 10 |
| 3 | GSK3ß | - | Phosphorylation | 3q13 | 267 | 5 | 2 | 1 | 8 | 4 |
| 4 | PPP3CA | PP3 | Dephosphorylation | 4q21 | 323.7 | 40 | 3 | 0 | 43 | 40 |
| 5 | CAST | Calpastatin | Degradation | 5q15 | 112.4 | 29 | 4 | 3 | 36 | 36 |
| 6 | PPP2CA | PP2A | Dephosphorylation | 5q31 | 28.8 | 2 | 3 | 1 | 6 | 5 |
| 7 | HSPA4 | HSP70 | Other | 5q31.1 | 53.05 | 4 | 1 | 1 | 6 | 3 |
| 8 | CSNK1A1 | CK1 | Phosphorylation | 5q32 | 56.16 | 10 | 1 | 1 | 12 | 11 |
| 9 | CAMK2A | CAMKA | Phosphorylation | 5q33 | 70.28 | 25 | 5 | 3 | 33 | 30 |
| 10 | HSPA1A | HSP70-1A | Other | 6p21 | 2.38 | 2 | 1 | 0 | 3 | 2 |
| 11 | FYN | - | Phosphorylation | 6q21 | 212.1 | 38 | 1 | 4 | 43 | 43 |
| 12 | WISP3 | - | Phosphorylation | 6q21 | 15.6 | 6 | 1 | 0 | 7 | 7 |
| 13 | PPP1R3A | PP1 | Deshosphorylation | 7q31 | 44.68 | 4 | 0 | 0 | 4 | 4 |
| 14 | CDK5 | - | Phosphorylation | 7q36 | 4.1 | 5 | 0 | 1 | 6 | 5 |
| 15 | PPP2R2A | PP2A | Dephosphorylation | 8p21.2 | 79.61 | 12 | 3 | 1 | 16 | 16 |
| 16 | WISP1 | - | Phosphorylation | 8q24 | 38.3 | 21 | 1 | 2 | 23 | 23 |
| 17 | MGEA5 | OGA | O-glcNAcylation | 10q24 | 33.97 | 4 | 1 | 1 | 6 | 5 |
| 18 | F2 | PT | Degradation | 11p11 | 20.3 | 2 | 2 | 1 | 2 | 2 |
| 19 | CTSD | - | Degradation | 11p15 | 11.24 | 3 | 1 | 1 | 3 | 3 |
| 20 | MARK2 | PAR1 | Phosphorylation | 11q12 | 70.15 | 7 | 5 | 4 | 7 | 7 |
| 21 | CAPN1 | - | Degradation | 11q13 | 30.13 | 8 | 2 | 2 | 8 | 7 |
| 22 | TTBK2 | - | Phosphorylation | 15q15 | 176.5 | 5 | 1 | 1 | 5 | 4 |
| 23 | MAPK3 | ERK1 | Phosphorylation | 16p11 | 9.2 | 4 | 2 | 2 | 4 | 2 |
| 24 | CDK5R1 | P35 | Phosphorylation | 17q11 | 4.17 | 6 | 2 | 1 | 6 | 6 |
| 25 | MAPT | Tau | - | 17q21 | 133.9 | 15 | 3 | 0 | 15 | 15 |
| 26 | PRKCA | PKCA | Phosphorylation | 17q22 | 507.9 | 8 | 2 | 1 | 9 | 9 |
| 27 | CSNK1D | HCKID | Phosphorylation | 17q25 | 29.33 | 4 | 1 | 0 | 4 | 4 |
| 28 | PRKACA | PKA | Phosphorylation | 19p13 | 26.05 | 4 | 1 | 1 | 4 | 3 |
| 29 | PIN1 | - | Other | 19p13 | 14.36 | 4 | 2 | 1 | 8 | 8 |
| 30 | MARK4 | - | Phosphorylation | 19q13 | 53.7 | 6 | 1 | 1 | 6 | 6 |
| 31 | CSNK2A1 | CKII | Phosphorylation | 20p13 | 61.15 | 11 | 2 | 1 | 11 | 11 |
| 32 | WISP2 | - | Phosphorylation | 20q12 | 12.6 | 3 | 1 | 3 | 3 | 3 |
| 33 | MAPK1 | ERK2 | Phosphorylation | 22q11 | 108 | 8 | 3 | 1 | 8 | 8 |
| 34 | OGT | - | O-glcNAcylation | Xq13 | 42.81 | 3 | 3 | 2 | 6 | 5 |
|  | **Total** |  |  |  |  | **323** | **67** | **44** | **384** | **355** |

| **Table S2. SNPs in *PPP3R1* associated with CSF ptau_181_ levels in the discovery series (WU-ADRC-CSF).** | | |
| --- | --- | --- |
| **rs** | **MAF** | **ptau_181_** |
| rs1868402^A^ | 0.37 | **5.90×10^-04^** |
| rs6546366^A^ | 0.35 | **0.0040** |
| rs1060842^A^ | 0.37 | **0.0005** |
| rs4671880 | 0.23 | **0.0080** |
| rs12713636^A^ | 0.36 | **0.0004** |
| rs13028330 | 0.23 | **0.0160** |
| rs1020824^A^ | 0.36 | **0.0003** |
| SNPs associated with CSF ptau_181_ levels after FDR correction are showed.. MAF= Minor Allele Frequency. A: Dominant model. B: Recessive model | | |
