# Supplementary material for: SNPs Associated with Cerebrospinal Fluid Phospho-Tau Levels Influence Rate of Decline in Alzheimer's Disease
Source: PLoS Genet. 2010 Sep 16;6(9):e1001101. doi: 10.1371/journal.pgen.1001101 (PMC2940763; doi:10.1371/journal.pgen.1001101)
Supplement: Table S2 — SNPs in PPP3R1 associated with CSF ptau181 levels in the discovery series (WU-ADRC-CSF). SNPs associated with CSF ptau181 levels after FDR correction are shown. MAF = Minor Allele Frequency. A: Dominant model. B: Recessive model. (0.03 MB DOC) [file pgen.1001101.s005.doc]

| **rs** | **MAF** | **ptau_181_** |
| --- | --- | --- |
| rs1868402^A^ | 0.37 | **5.90×10^-04^** |
| rs6546366^A^ | 0.35 | **0.0040** |
| rs1060842^A^ | 0.37 | **0.0005** |
| rs4671880 | 0.23 | **0.0080** |
| rs12713636^A^ | 0.36 | **0.0004** |
| rs13028330 | 0.23 | **0.0160** |
| rs1020824^A^ | 0.36 | **0.0003** |
